# Rodent-avoidance, topography and forest structure shape territory selection of a forest bird

**DOI:** 10.1186/s12898-016-0078-8

**Published:** 2016-05-09

**Authors:** Gilberto Pasinelli, Alex Grendelmeier, Michael Gerber, Raphaël Arlettaz

**Affiliations:** Swiss Ornithological Institute, Sempach, Switzerland; Department of Evolutionary Biology and Environmental Studies, University of Zurich, Zurich, Switzerland; Division of Conservation Biology, Institute of Ecology and Evolution, University of Bern, Bern, Switzerland; Schweizer Vogelschutz SVS/BirdLife Schweiz, Zurich, Switzerland; Swiss Ornithological Institute, Valais Field Station, Sion, Switzerland

**Keywords:** Habitat, Ecological niche, Forestry, AICc model selection, Aves, Passeriformes

## Abstract

**Background:**

Understanding the factors underlying habitat selection is important in ecological and evolutionary contexts, and crucial for developing targeted conservation action in threatened species. However, the key factors associated to habitat selection often remain poorly known. We evaluated hypotheses related to abiotic and biotic factors thought to affect territory selection of the wood warbler *Phylloscopus sibilatrix*, a passerine living in an unpredictable environment owing to irregular rodent outbreaks and showing long-term declines particularly in Western Europe.

**Results:**

Comparing breeding territories to unoccupied areas located close-by revealed that territory occupancy in north-western Switzerland was positively related to slope steepness (topographic hypothesis supported) as well as to numbers of tussocks and trees, respectively, while it showed a unimodal relationship to cover of herb layer (forest structure hypothesis supported). Furthermore, a strong negative correlation between breeding territory occupancy and rodent numbers was found, suggesting that wood warblers avoid areas with high rodent densities (rodent-avoidance hypothesis supported). Comparing breeding territories to abandoned territories showed that breeding territories were located on steeper slopes (topography hypothesis supported), at larger distance from the forest edge (anthropogenic disturbance hypothesis supported) and harboured more trees (forest structure hypothesis supported) than abandoned territories.

**Conclusions:**

Aside from structural and topographic features of the habitat, wood warblers are affected by rodent numbers when settling, making habitat selection unpredictable from year to year. Forestry practices promoting relatively high tree densities, few bushes and an intermediate low-growing ground vegetation cover would enhance habitat quality for this declining passerine. In contrast, forestry practices aiming at increasing light in forests (selective thinning, group-felling) or keeping forest stands permanently covered with shrubs, bushes and trees of various sizes (continuous cover forestry) do not benefit the wood warbler.

**Electronic supplementary material:**

The online version of this article (doi:10.1186/s12898-016-0078-8) contains supplementary material, which is available to authorized users.

## Background

Understanding the mechanisms underlying habitat selection is important in ecological and evolutionary contexts as well as for the application of conservation measures in threatened species. For animals reproducing in seasonal environments, selecting a habitat to breed is a recurring annual task. Resident species can base breeding habitat selection on year-round interactions with their abiotic and biotic environments. In contrast, long-distance migratory species such as many songbirds, often spending most of the year outside the breeding grounds, have to select habitats shortly after returning to the breeding grounds. Here, we address patterns of habitat selection of the wood warbler *Phylloscopus sibilatrix*, a specialised songbird inhabiting the interior of European woodlands. These woodlands are subject to irregular rodent outbreaks arising from irregularly occurring seed mast of forest trees [1]. Wood warblers have been shown to respond to rodent numbers when settling in spring [2, 3], but how abiotic factors, structural habitat features and rodent numbers interact during habitat selection of this species has not yet been assessed.

We examined hypotheses proposed to be relevant both in the general context of habitat selection and in explaining population declines of the wood warbler in Western Europe. We compared current breeding territories to both unoccupied areas located nearby and to abandoned territories, that is, to previously but no longer occupied territories (see “Choice of abandoned territories”). According to the forest structure hypothesis, structural changes such as decreases in canopy cover or increases in understory vegetation over the last decades [e.g., 4] may have resulted in degradation and loss of many previously suitable forest habitats [5]. In contrast, in ecosystems like Białowieża National Park in Poland, a pristine environment mostly unaffected by humans, behaviour, ecology, breeding success and population trends of wood warblers did not significantly change over the past 25 years [2, 6]. While macro-habitat selection of the wood warbler has been subject to some studies [7–9], factors affecting habitat selection at the territory scale have received comparatively little attention [5, 10–12]. Based on habitat preferences established in previous studies, we expected breeding territories of wood warblers to be located in forest stands of medium age and to be characterized by relatively closed canopy, sparse undergrowth and sparse ground vegetation cover compared to unoccupied control areas and abandoned territories, respectively.

Predation risk has been shown to affect patterns of habitat selection in a variety of species [13–15]. In birds, nest predation is often the main reason for nest failure [16, 17] and can profoundly affect avian population dynamics. Predation has been shown to cause up to 95 % of all nest losses in the wood warbler [e.g., 6]. As suggested for other bird species [18, 19], avoiding areas of increased predation risk should thus be of central importance in breeding habitat selection of this species. Many potential wood warbler nest predators such as red fox (*Vulpes vulpes*) and marten (*Martes* spp.) feed on rodent species such as voles (*Microtus* spp.) and mice (*Apodemus* spp.). Increased density and activity of these predators in areas and years with high rodent populations might increase predation risk for wood warbler nests, in addition to the possible risk of direct predation by rodents. Numbers of wood warblers are lower in rodent outbreak years than in other years [2, 3]. High rodent density might indicate increased predation risk to wood warblers, which may thus avoid settling in such areas. We examined the hypothesis that wood warbler habitat selection at the territory scale was related to rodent density. According to this rodent-avoidance hypothesis, we expected that actual territories of wood warblers would have lower rodent densities than nearby unoccupied areas and that abandoned territories (see “Choice of abandoned territories” below) would show higher rodent densities than current breeding territories.

Disturbances due to increasing human activities can negatively affect breeding bird communities and population dynamics [20–23] and have been proposed to be a reason for wood warbler population declines in Switzerland [24]. Therefore, we evaluated the influence of disturbance-related variables on breeding and abandoned territories (referred to in the following as the “anthropogenic disturbance hypothesis”), expecting that abandoned territories would be located closer to areas exposed to human disturbance than breeding territories.

Finally, abiotic factors are part of a species’ niche and are thus expected to affect habitat selection directly or indirectly [e.g. 25]. Accordingly, wood warblers have been found to prefer settling in inclined areas [5, 7, 26]. We thus expected breeding territories to be located on steeper slopes than control areas. Additionally, a preference for slopes with eastern to southerly aspects has been reported, while slopes with western and northern aspects appear to be avoided, this preference remaining unexplained [7, 26]. Breeding territories were therefore expected to exhibit more eastern to southern aspects than control areas. Because the wood warbler has disappeared from many parts of the Swiss lowlands, we furthermore expected breeding territories, compared to abandoned territories, to be located at higher elevation, on steeper and more east- to south-exposed slopes. The latter relationship was expected as wood warblers might today be restricted to the best available sites, i.e. to the most suitable aspects. Slope steepness, aspect and elevation (m above sea level, a.s.l.) are referred to as the topography hypothesis.

Throughout Western Europe, populations of this species have declined in the last three decades, while populations in Eastern Europe have remained relatively stable [6, 7, 27]. The causes of these differential population trajectories are unknown. The wintering grounds in tropical Africa do not appear to differ for birds from western and eastern populations [28], suggesting that changes in the breeding areas could underlie the diverging population trends. In Switzerland, the wood warbler is classified as vulnerable (VU) on the red list of breeding birds [29] and considered a priority species for the Swiss species recovery programme for birds [30]. Aside from evaluating habitat selection under unpredictable environmental conditions arising from irregular rodent outbreaks, an additional aim was thus to increase our understanding of the habitat requirements of this species to provide conservationists and foresters with management recommendations to ameliorate the species’ habitat.

## Methods

### Study species

The wood warbler is a Eurasian, ground-nesting passerine wintering in tropical Africa and returning to the breeding grounds in mid-April. Unpaired males use a characteristic singing style and show a high singing activity from early morning throughout the day. After pairing, the singing style changes, and overall singing activity sharply drops to relatively low levels for the rest of the breeding season [7]. The changes in singing style use and singing activity allows distinguishing paired males from unpaired males.

### Study areas

**Fig. 1:**
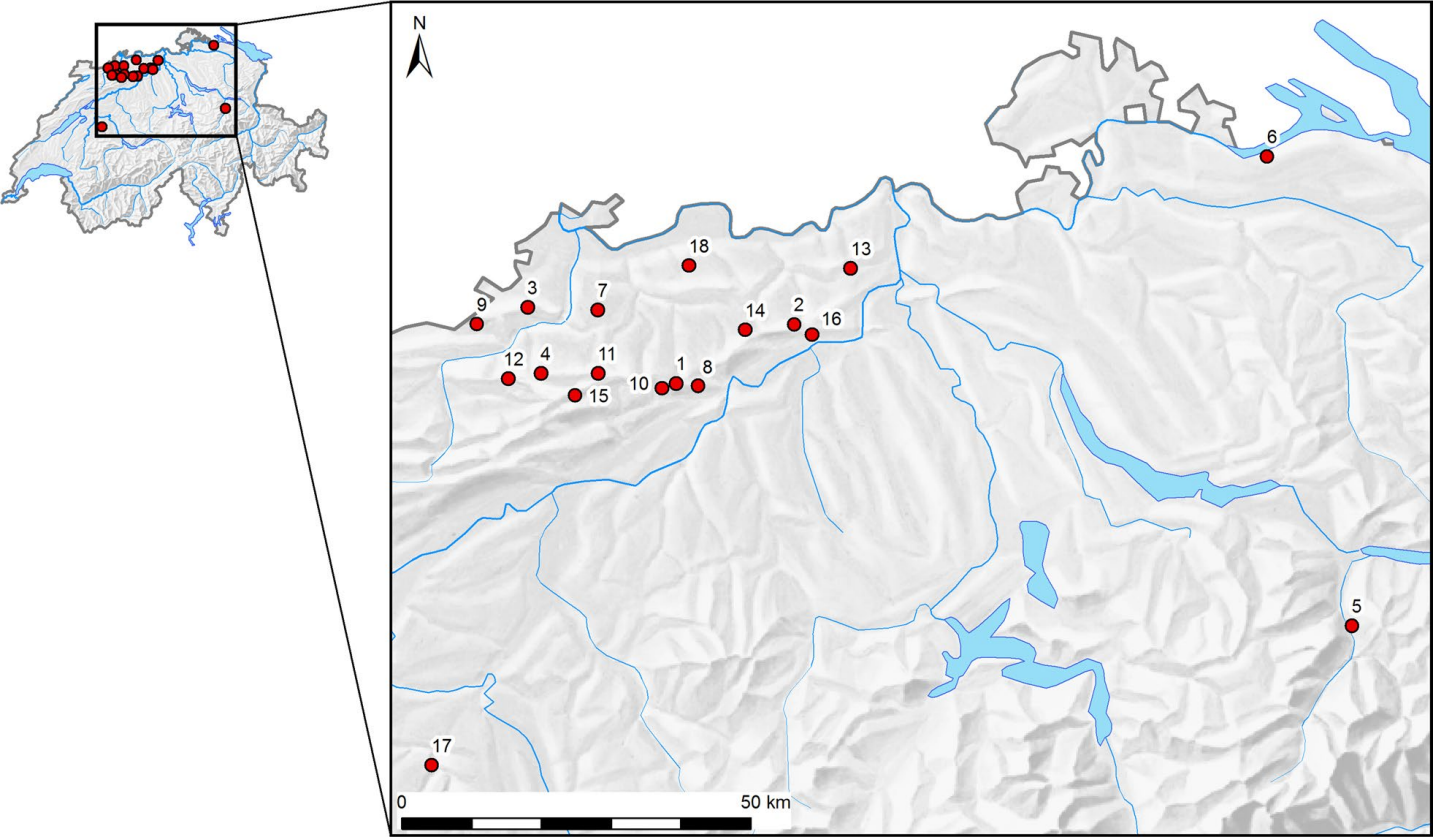
Location of study areas in Switzerland. 1 = Belchen, 2 = Bänkerjoch, 3 = Blauen, 4 = Erschwil, 5 = Ennenda, 6 = Gündelhardt, 7 = Hochwald, 8 = Homberg, 9 = Kleinlützel, 10 = Langenbruck, 11 = Lauwil, 12 = Montsevelier, 13 = Mönthal, 14 = Oltingen, 15 = Scheltenpass, 16 = Staffelegg, 17 = Ueberstorf, 18 = Wintersingen. Study sites 13, 17 and 18 only used in the comparison between breeding territories vs. abandoned territories. See Additional file 1 for details on the study areas. Basemap © Institute of Cartography and Geoinformation, ETH Zurich, reproduced with permission from 11 April 2016

We searched for wood warbler territories in 15 study areas (Fig. 1; Additional file 1), which were chosen based on (a) the common breeding bird survey provided by the Swiss Ornithological Institute (the standardized Swiss national bird monitoring program, http://www.vogelwarte.ch/monitoring-common-breeding-birds.html), (b) the breeding bird atlas of the canton Zurich (www.birdlifezuerich.ch) and (c) http://www.ornitho.ch/ (the official birding exchange platform in Switzerland). In all study areas, we used the coordinates of previous sightings as rough starting points, from where we extensively searched for the species. The boundaries of the study areas were determined by natural circumstances like forest edge, strong changes in forest structure (e.g. young re-growths or coniferous plantations) or geographical features (e.g. deep valleys). Study areas were mostly located on steep, south-facing slopes within large deciduous forests dominated by beech *Fagus silvatica* and occasionally oaks *Quercus* spp. pine *Pinus silvestris* and fir *Picea abies* were intermixed to various degrees. No permissions were required to enter the forests. Size of the study areas is given in Additional file 1.

### Territory mapping and nest searching

We started mapping territories in mid-April 2010–2012 by listening for the distinct wood warbler song. If no wood warbles were heard or observed, we played wood warbler songs for 10 s every 300 m to avoid overlooking territories. As soon as birds responded, we stopped the playback and noted the observations in a map. We checked each study area for the presence of wood warblers at least once a week until early July. A territory was classified as occupied when (a) we observed a singing male twice in the same location with at least 7 days in between, (b) we observed a pair (two birds in the same territory showing no agonistic behaviour and observed at least twice on subsequent visits) or (c) we found the nest. Once territories had been established, they were regularly checked for the presence of females and nests. Due to the regular observer presence, a narrow search grid and the use of playback, it is highly probable that all territories (with and without nests) within a study area were found. Once the nest site was established, singing and general activity of both males and females concentrated to a small radius (mostly <25 m) around the nest, which corresponds to a circular area of about 1900 m^2^, representing the upper end of the average breeding territory size after nest establishment [7]. For logistical reasons, it was impossible to record habitat variables and rodent density for all breeding territories. Therefore, territories were selected to get a representative number of successful and unsuccessful nests and a balanced sample from the different study areas. As we were interested in the patterns of habitat selection in breeding territories, territories without nests were not considered for habitat mapping.

### Choice of control areas

To each breeding territory chosen for analysis we assigned a control area without wood warblers. We first defined eight possible control areas (i.e. X–Y-coordinates) 200–300 m from the nest of the respective territory in the four cardinal (N, E, S, W) and four inter cardinal (NE, SE, SW and NW) directions. To avoid trivial results, we ruled out control areas with habitats known to be unsuitable for wood warblers (non-forest areas, large clearings, purely coniferous forest patches, young re-growths and plantations). Also, control areas closer than 50 m to other breeding territories were excluded. This distance was based on the average breeding territory size (1200–1900 m^2^) according to [7]. Of the non-excluded potential control areas, one was randomly selected. Absence of wood warblers in retained control areas was confirmed with playback.

### Choice of abandoned territories

Abandoned territories were selected based on patterns of wood warbler occupancy over the past 10 years. Abandoned territories were defined as areas that had been deserted for the last 3 years, but that had been occupied at least three times in the 7 years before. This ensured that now-abandoned territories had not simply been in marginal habitats when they were occupied earlier and also accounted for the known nomadic behaviour of the species [2]. Based on data from the common breeding bird survey of the Swiss Ornithological Institute, we selected and analysed 20 abandoned territories in 6 study areas (Additional file 1). This relatively low number of abandoned territories was a consequence of the need to know the exact location of the territory for recording habitat variables (see “Habitat variables” below). The centre of an abandoned territory was defined as the X–Y-coordinate averaged over the mapped observations (accuracy ~50 m) made during the three annual surveys of the Swiss common breeding birds monitoring scheme.

### Habitat variables

Habitat variables were recorded after a nest was lost (predated or abandoned) or the nestlings had fledged to minimize disturbance at active nests. In the control areas, habitat variables were recorded at the same time as in their associated breeding territories. In abandoned territories, habitat variables were sampled towards the end of the breeding season.

**Fig. 2:**
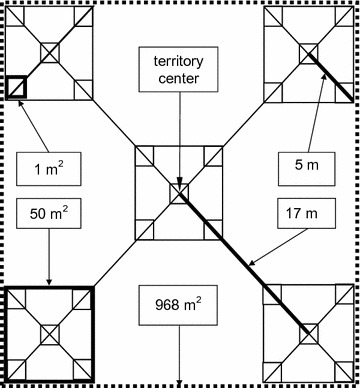
Sampling design for assessing habitat structure variables and rodent numbers. Territory center is the nest position in occupied territories or X/Y-coordinates in control areas and abandoned territories (see text for details). *Bold lines* indicate distances, *bold squares* exemplify 1 m^2^ and 50 m^2^ squares, respectively. Adapted from [42]

For each breeding territory, control area and abandoned territory, respectively, we defined five quadratic sample areas of 50 m^2^ each, as shown in Fig. 2. One square was centred on the territory centre (nest position, X/Y-coordinates in control areas and abandoned territories, see above), the centres of the other four squares were located 17 m from the territory centre on axes corresponding to the diagonals of the first square. Connecting the outermost points of these four squares results in an area covering roughly 1000 m^2^ (Fig. 2), which corresponds to the lower end of the average breeding territory size of wood warblers [7]. Furthermore, we defined five 1-m^2^-squares located at the corners and the centre, respectively, in each of the five 50 m^2^-squares (Fig. 2).

Habitat characteristics were described with variables referring to forest structure, rodent abundance, anthropogenic disturbance and topography. Names, descriptions, calculations and values of variables are listed in Table 1. *Aspect*, a circular variable, was converted to the variables *southness* [−cos(*aspect* × π/180), where 1 = S and −1 = N] and *eastness* [sin(*aspect* × π/180), where 1 = E and −1 = W].

**Table 1:** Variable names and descriptions and associated hypotheses

Hypothesis	Variable	Description	Breeding	Control	Abandoned
Forest structure					
Ground variables	Cover of herb layer ^a^	Percentage of ground covered by vegetation < 0.5 m, visually estimated	24.2, 10.9–42.6	14.8, 6.4–32.0	25.5, 12.2–29.2
	Number of tussocks ^b^	Number of grass and sedge tussocks	325.5, 122.8–653.5	40.0, 3.0–216.0	28.5, 0–310.8
	Number of bushes ^c^	Number of bushes > 0.5 m height and number of young trees with dbh < 8 cm	38.5, 17.8–70.8	69.0, 12.0–246.0	34.0, 6.8–137.8
Tree variables	Number of trees ^c^	Number of trees with dbh > 8 cm	16.5, 13.0–22.0	13.0, 10.0–17.0	11.0, 7.8–14.5
	Number of trees branched < 4 m ^c^	Number of trees with branches below 4 m	10.0, 6.0–14.0	7.0, 4.0–11.0	5.5, 3.8–10.3
	Number of trees branched < 10 m ^c^	Number of trees with branches below 10 m	13.0, 9.0–18.0	9.0, 6.0–13.0	7.0, 6.0–12.0
	Tree dbh ^d^	Average dbh of all trees with dbh > 8 cm	26.0, 22.3–30.0	31.0, 26.0–36.0	27.5, 24–37.5
	Tree species diversity ^e^	Shannon’s index of diversity based on tree species and dbh data	1.2, 0.7–1.5	0.9, 0.7–1.3	0.9, 0.6–1.2
	Sky visibility ^d^	Percentage of sky visible (see “Estimation of sky visibility” section)	13.0, 9.0–19.0	14.0, 10.0–19.0	10.5, 9.0–21.8
	Proportion beech ^e^	Number of beech trees divided by total number of trees	43.2, 20.3–59.3	50.0, 29.2–69.6	52.3, 33.3–58.5
	Proportion other deciduous trees ^e^	Number of deciduous trees except beech divided by total number of trees	31.7, 18.6–47.1	25.0, 14.3–50	41.4, 18.6–53.1
	Proportion conifers ^e^	Number of coniferous trees divided by total number of trees	13.1, 0–31.7	0, 0–29.4	0, 0–15.4
Rodent-avoidance	Rodent numbers	Number of rodents captured in the 25 traps per territory or control area	8.0, 0–15.8	13.0, 4.0–22.0	7.0, 1.0–13.3
Anthropogenic disturbance	Distance to paths ^f^	Distance to paths, i.e. trails regularly used by humans	48.0, 15.0–75.8	–	37.5, 25.8–45.5
	Distance to forest edge ^f^	Distance to edge of forest	148.5, 102.8–237.8	153.0, 72.0–224.0	98.5, 60.8–148.5
Topography	Elevation ^f^	Elevation in m above sea level	698, 656–931	699, 610–920	575, 548–740
	Aspect ^d^	Measured in degrees (^o^) with a compass in the centre of each 50-m^2^-square	174, 144–204	171, 127–227	162, 109–307
	Slope steepness ^d^	Measured in degrees (^o^) with a compass in the centre of each 50-m^2^-square	31.5, 27.0–37.0	26.0, 21.0–33.0	21, 16.8–23.5

Shown are medians and interquartile (25–75 %) ranges

Dbh = diameter at breast height. N = 73 for breeding territories and control areas, respectively, and n = 20 for abandoned territories

^a^ Averaged over the 25 1-m^2^-squares per breeding territory, per control area and per abandoned territory, respectively

^b^ Summed over the 25 1-m^2^-squares per breeding territory, per control area and per abandoned territory, respectively

^c^ Summed over the five 50-m^2^-squares per breeding territory, per control area and per abandoned territory, respectively

^d^ Averaged over the five 50-m^2^-squares per breeding territory, per control area and per abandoned territory, respectively

^e^ Calculated over the five 50-m^2^-squares per breeding territory, per control area and per abandoned territory, respectively

^f^ Recorded for the centres of each breeding and abandoned territory and extracted from ecoGIS (www.ecogis.admin.ch)

#### Estimation of sky visibility

The percentage of sky visible from 1.5 m above ground level at each of the five 50 m^2^-squares was recorded from pictures of the crown canopy in order to estimate foliage density. We adopted the method described in [31], with the following adaptions and additions. We used a DSLR camera (Nikon D2Xs) with a standard zoom lens (18–70 mm f3.5-4.5G ED-IF AF-S DX Zoom Nikkor) at a focal length of 35 mm. To take a picture, the camera was held 1.5 m above ground in the centre of the respective 50 m^2^-square, lens pointing vertically up. Pictures were taken in camera RAW format and imported into Adobe Photoshop CS5 for editing. Import was performed with standard camera RAW settings. Brightness of green colours was reduced to the minimum and brightness of blue colours increased to the maximum in order to improve the contrast between sunlit green leaves and the blue sky. The pictures were edited as described in [31], downscaled to 1500 × 1000 pixels and transformed to black/white bitmaps before being processed by a self-written php-script to calculate the black/white pixel ratio.

#### Live-trapping of rodents

Rodents were captured with live-trapping in breeding territories, control areas and abandoned territories with permission nr. 410 issued by the Veterinary Office of the Canton Basel-Landschaft. For details about laws on animal experimentation in Switzerland see http://www.blv.admin.ch/themen/tierschutz/00777/index.html?lang=en. To avoid disturbance and for logistic reasons, we waited until at least three nests per study area had failed and/or had fledged. We used the same sampling layout for rodent trapping as for recording habitat variables (Fig. 2). In each of the five 50 m^2^-squares, five traps were placed near structures or, if found, near rodent trails, and covered with foliage. Thus, 25 traps were set up in each breeding territory, control area and abandoned territory, respectively. We used Longworth traps (Penlon Ltd., Abingdon, UK) made of steel or aluminium and Field Trip Trap Live Catch Trap made of plastic (Alana Ecology, Bishops Castle, UK). We provided commercial pet hay as bedding and apple pieces, oatmeal, peanut butter and hazel nuts as bait.

Traps were put out on the same day in a breeding territory and in its associated control area. Traps were active during 48 h. In 2010, traps were checked every 8 h, resulting in five capture occasions. Based on experiences from 2010, traps were checked every 12 h in 2011 and 2012, resulting in three capture occasions. Caught animals were put into a bag, classified to species or genus level (cryptic sibling species), marked by hair clipping and released immediately. We marked the animals using a nose hair trimmer, with the markings reflecting each capture occasion. From these markings, capture histories were later constructed to allow calculation of capture probabilities and rodent density.

### Statistical analyses

#### Estimation of rodent density and rodent numbers

We analysed capture-recapture data using Program CAPTURE run through Program MARK v6.0 [32] and assumed a demographically closed population, since our trapping time frame only lasted 48 h. Even though we caught and identified several species, we pooled all captures to obtain a single estimate of rodent density per breeding territory, control area and abandoned territory, respectively. With Program CAPTURE, we computed estimates of capture probability and population density for the following models. (1) A null model of no time, behavior or heterogeneity effect (Mo), assuming all individuals of a population are equally at risk of capture on every trapping occasion. (2) A model of heterogeneity effects (Mh), assuming capture probabilities vary by individual animal. (3) A model with time effects (Mt), assuming capture probabilities vary with time. (4) A model of behavior effects (Mb), assuming capture probabilities vary by behavioral response to capture. (5) A model of behavior and heterogeneity effects (Mbh), assuming capture probabilities vary by individual animal and by behavioral response to capture. (6) A model of time and heterogeneity effects (Mth), assuming capture probabilities vary with time and by individual. (7) A model of time and behavior effects (Mtb), assuming capture probabilities vary with time and with behavioral effects (trap happiness, trap shyness). (8) A model of time, heterogeneity and behavior effects (Mtbh), for which however, there is currently no estimator. The first 7 models were then ranked by a model selection criterion between 0 and 1, where the most appropriate model scores a 1. We then used the rodent density estimate from the most appropriate model, calculated for each breeding territory, control area or abandoned territory for further analyses.

In addition to rodent density, we calculated the total number of trapped rodents by summing all captures over the 25 traps per territory, control area and abandoned territory, respectively (“rodent numbers”). Rodent density and rodent numbers turned out to be highly correlated (Spearman rank correlation, r_s_ = 0.97, n = 131). In the following, we only used rodent numbers, because estimation of rodent density was not possible for all territories in 2011 owing to very few rodent captures (rodent crash year).

#### Correlations among habitat variables

Strong (Spearman rank correlation coefficient ∣r_s_∣ > 0.7) and positive correlations were only detected between the variables *number of trees* and *number of trees branched* <*4* *m*, between *number of trees* and *number of trees branched* <*10* *m*, and between *number of trees branched* <*4* *m* and *number of trees branched* <*10* *m* (see Additional file 2). In the data set used for comparing breeding territories and abandoned territories, two additional strong (and negative) correlations were found between the variables *tree dbh* (diameter at breast height) and *number of trees branched* <*4* *m*, and between *proportion beech* and *proportion other deciduous trees*, respectively. In all subsequent analyses, we therefore never entered both variables of a strongly correlated variable pair into the same generalized linear mixed-effects model.

#### Model structure

Generalized linear mixed-effects models (GLMMs) with logit link and binomial error structure were used to assess the relationships between breeding territory selection and the habitat variables. The binary dependent variable always was the occupancy state of a site (0 for control areas and abandoned territories, respectively; 1 for breeding territories). The habitat variables potentially influencing breeding territory selection were defined as fixed effects. For the analysis of breeding territories vs. control areas, we used random effects with a hierarchical structure. The breeding territories were distributed over different study areas. Within the study areas, breeding territories and their control areas were always paired. Thus, we included two random effects: (1) *study area* to account for the dependency of breeding territories within the same study area and (2) *breeding territory*-*control area pairs nested within study area* to account for the paired structure of breeding territories and control areas. Given the nomadic behaviour and low philopatry of the wood warbler [2; own unpublished ringing data], the chance of observing the same individuals over multiple years was negligible, making the inclusion of a random effect to account for individual dependencies unnecessary. Likewise, locations of nest sites changed across years, so breeding territories were not repeatedly sampled over time. For the analysis of abandoned vs. breeding territories, we used no random effects for two reasons. (1) Abandoned territories were available from only 3 of the 15 study areas used to compare breeding territories vs. control areas and from 3 additional study areas (Additional file 1). (2) Abandoned territories were often located quite far away from breeding territories of the same study area, sometimes even in other forest stands; therefore, using a paired structure in the statistical analysis was not expedient.

Prior to the analyses, variables (all continuous) were standardized (mean = 0, standard deviation = 1). Analyses were performed in R (https://www.r-project.org/) using the packages lme4 [33] for model selection and AICcmodavg [34] for model-averaging. Note that model-averaged coefficients were very similar to those of the respective best across-hypothesis models [cf. 35]. Model fit was visually assessed with residual plots. Further, we evaluated the presence of spatial autocorrelation with semi-variograms of the residuals [36]. Evidence for spatial autocorrelation was found in the analysis of breeding territories vs. abandoned territories, but not in the analysis of breeding territories vs. control areas. We thus included x- and y-coordinates of the territories and the interaction between x- and y-coordinates in all analyses of breeding territories vs. abandoned territories to account for spatial structure. Inspection of semi-variograms of the residuals following these analyses no longer indicated presence of spatial autocorrelation.

#### Modeling approach and model selection

**Fig. 3:**
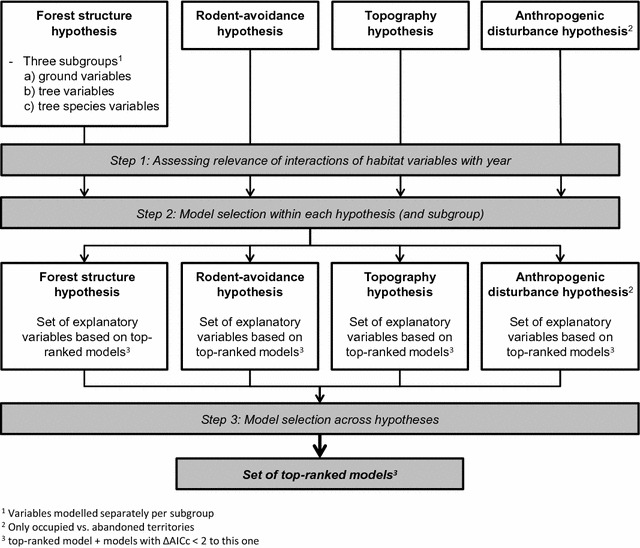
Overview on the model selection design applied. Variables of the forest structure hypothesis were placed in three thematic subgroups to avoid over-parameterizing and convergence problems of models. For further details, see “Modeling approach and model selection” section and Additional file 3

The forest structure hypothesis included 12 habitat variables. To avoid over-parameterizing and convergence problems of models, variables of the forest structure hypothesis were assigned to three subgroups termed (1) “ground variables”, which included *cover of herb layer*, *number of tussocks*, *number of bushes*; (2) “tree variables”, which included *number of trees*, *number of trees branched* <*4* *m*, *number of trees branched* <*10* *m*, *tree dbh*, *tree species diversity* and *sky visibility*; and (3) “tree species composition”, which included *proportion beech*, *proportion other deciduous trees* and *proportion conifers*. Models consisting of variables from each of these three subgroups were then separately evaluated (i.e. variables from the different subgroups were not jointly modelled in the first two steps, Fig. 3). The rodent-avoidance hypothesis only included the variable *rodent numbers*, the anthropogenic disturbance hypothesis (only evaluated for breeding vs. abandoned territories), *distance to paths* and *distance to forest edge*. The topography hypothesis included the variables *slope steepness, southness* and *eastness* (for *aspect*, see above) and, in the comparison of breeding vs. abandoned territories, *altitude* (Table 1). *Southness* and *eastness* were always jointly entered to or removed from models.

Candidate models (see Additional file 3) were compared with Aikake’s Information Criterion corrected for small sample size AICc [37]. Models were ranked based on their AICc values, with the model having the lowest AICc being considered the best, given the data. Candidate models were evaluated as follows: models with ΔAICc < 2 compared to the best model were judged to have considerable support by the data. Competing models with ΔAICc < 2 compared to the best model, but differing by one parameter only, were evaluated with regard to their log-likelihood value. If the log-likelihood of a model containing the habitat variable A was almost equal to a model including A and habitat variable B, then B did not explain much additional variation in the data [37], and the model with both A and B was discarded in favour of the model with A only.

## Results

We only report results for analyses with *number of trees*. Analyses including *number of trees branched* <*4* *m* or *number of trees branched* <*10* *m*, both highly correlated to *number of trees* (see Additional file 2), gave almost identical results to analyses with *number of trees* and are presented in Additional file 4.

### Breeding territories vs. control areas

#### Interactions with year (analysis step 1)

In the forest structure hypothesis, subgroup ground variables, two models including either the interaction of *cover of herb layer* and *year* or *number of bushes* and *year* were ranked highest and received very similar support (ΔAICc between models = 0.147). ΔAICc of the null model to the best model was 36.777. In the rodent-avoidance hypothesis, the model including the interaction between *rodent numbers* and *year* was ranked highest, with the null model having a ΔAICc of 5.057 to the highest ranking model. In the other hypotheses (and subgroups), models with interactions with *year* generally received low support (ΔAICc always >4.3 to best models). We thus retained the interactions of *cover of herb layer* and *year*, *number of bushes* and *year* and *rodent numbers* and *year* for the next step.

#### Within-hypothesis analysis (analysis step 2)

##### Forest structure hypothesis

Subgroup ground variables—Evaluation of models with all possible combinations of habitat variables showed that the model including *number of bushes*, *number of tussocks* and the quadratic effect of *cover of herb layer* was ranked highest, with ΔAICc of this model to the null model being >39 (Table 2). Three other models were within ΔAICc of 2 to the best model (details in Table 2). However, the fourth-ranked model including *number of bushes* and the quadratic effects of *number of tussocks* and *cover of herb layer* had almost the same log-likelihood value as the top model (Table 2). The quadratic effect of *number of tussocks* did thus not explain more variation in the data than the linear effect of *number of tussocks*. In summary, we retained *number of bushes*, *number of tussocks*, the quadratic effect of *cover of herb layer* and the interaction of *number of bushes* and *year* for the subsequent across-hypotheses analysis.

**Table 2:** Model selection results of the analysis of breeding territories vs. control areas (n = 73 pairs)

Hypothesis	Variables in model	LL	K	AICc	ΔAICc	Weight
Forest structure						
(a) Ground variables	Number of bushes, number of tussocks, cover of herb layer^2^	−77.238	7	169.288	0	0.232
	Number of bushes, year, number of bushes x year, number of tussocks	−75.155	9	169.633	0.345	0.195
	Number of bushes, number of tussocks	−80.208	5	170.845	1.557	0.106
	Number of bushes, number of tussocks^2^, cover of herb layer^2^	−77.089	8	171.230	1.942	0.088
	…					
	Null	−101.199	3	208.568	39.280	0.000
(b) Tree variables	Number of trees, tree dbh	−87.397	5	185.224	0	0.158
	Number of trees, tree dbh, tree species diversity^2^	−85.854	7	186.520	1.297	0.083
	Number of trees, tree dbh, tree species diversity	−87.160	6	186.924	1.701	0.068
	…					
	Null	−101.199	3	208.568	23.345	0.000
(c) Tree species composition	Null	−101.199	3	208.568	0	0.114
	Proportion beech, propoprtion other deciduous trees, proportion conifers^2^	−97.099	7	209.01	0.442	0.091
	Proportion beech, propoprtion other deciduous trees, proportion conifers	−98.227	6	209.059	0.491	0.089
	Proportion beech	−100.459	4	209.202	0.634	0.083
	Proportion beech^2^, propoprtion other deciduous trees, proportion conifers^2^	−96.542	8	210.134	1.566	0.052
Rodent-avoidance	Rodent numbers, year, rodent numbers x year	−93.230	8	203.511	0	0.498
	Rodent numbers	−98.100	4	204.483	0.972	0.306
	Null	−101.199	3	208.568	5.057	0.040
Topography	Slope steepness	−91.564	4	191.412	0	0.558
	…					
	Null	−101.199	3	208.568	17.156	0
Across hypotheses	Slope steepness, rodent numbers, number of tussocks, cover of herb layer^2^, number of trees, number of bushes, tree dbh	−62.749	11	149.469	0	0.107
	Slope steepness, rodent numbers, number of tussocks, cover of herb layer^2^, number of trees, number of bushes	−63.958	10	149.545	0.076	0.103
	Slope steepness, rodent numbers, number of tussocks, cover of herb layer^2^, number of trees, tree dbh	−64.066	10	149.762	0.293	0.092
	Slope steepness, rodent numbers, number of tussocks, cover of herb layer^2^, number of trees	−65.448	9	150.220	0.751	0.073
	Slope steepness, number of tussocks, cover of herb layer^2^, number of trees, number of bushes	−65.976	9	151.275	1.806	0.043
	Slope steepness, rodent numbers, number of tussocks, cover of herb layer^2^, number of trees, tree dbh, tree species diversity^2^	−62.470	12	151.285	1.816	0.043
	Slope steepness, rodent numbers, number of tussocks, cover of herb layer^2^, number of trees, tree species diversity^2^	−63.658	11	151.287	1.817	0.043
	…					
	Null	−101.199	3	208.568	59.099	0.000

For each hypothesis, the top-ranked model (ΔAICc = 0), the models with ΔAICc < 2 to the top-ranked model and the null model (referred to as “null”) are shown. “…” refers to additional models examined, but not listed in detail to avoid overlong table, as they were little informative

The quadratic effect of a variable x, composed of a linear and a quadratic component (x ± x^2^), is denoted as x^2^

*LL* log-likelihood, *K* number of parameters in the model (including random effects and intercept), *weight* Akaike weight (chance of the model to be the best one, given the candidate models)

Subgroup tree variables—The highest-ranked model included *number of trees* and *tree dbh* (ΔAICc of 23.34 to the null model, Table 2). Second-ranked was a model additionally including the quadratic effect of *tree species diversity*. The third-ranked model included the linear effect of *tree species diversity*, in addition to *number of trees* and *tree dbh.* However, log-likelihood of the third-ranked model was almost the same as for the highest-ranked model, suggesting that the inclusion of the linear effect of *tree species diversity* did not really contribute to explaining variation in the data. We thus retained *number of trees*, *tree dbh* and the quadratic effect of *tree species diversity* for the across-hypotheses analysis.

Subgroup tree species composition variables—The null model was ranked highest (Table 2). We thus did not retain any variable relating to tree species composition for the across-hypotheses analysis.

##### Rodent-avoidance hypothesis

The model including the interaction between *rodent numbers* and *year* was ranked highest, followed by the model with *rodent numbers* only (Table 2). We thus retained both *rodent numbers* and the interaction between *rodent numbers* and *year* for the across-hypothesis analysis.

##### Topography hypothesis

The model including *slope steepness* was ranked highest and the only one with support (ΔAIC to the next best model = 2.1, Table 2). We thus retained *slope steepness* for the across-hypotheses analysis.

#### Across-hypotheses analysis (analysis step 3)

Models including habitat variables retained from step 2 and their interactions with rodent numbers had ΔAICc > 2.2 to the highest-ranking model, which included the main effects *number of tussocks* and *rodent numbers* only (see Additional file 3 for explanation of modelling steps). Interactions with *rodent numbers* were thus not further considered.

The highest-ranked model (ΔAICc of 59.1 to the null model, Table 2) included the following variables: *slope steepness* (topography); *rodent numbers* (rodent avoidance); and, within forest structure: *number of tussocks*, *number of bushes* and the quadratic effect of *cover of herb layer* (subgroup ground variables); *number of trees* and *tree dbh* (subgroup tree variables). Six other models had ΔAICc values <2 to the highest-ranked model; they all included *slope steepness,number of tussocks*, *number of trees* and the quadratic effect of *cover of herb layer*. Five of the six high-ranking models further included *rodent numbers* (Table 2).

**Fig. 4:**
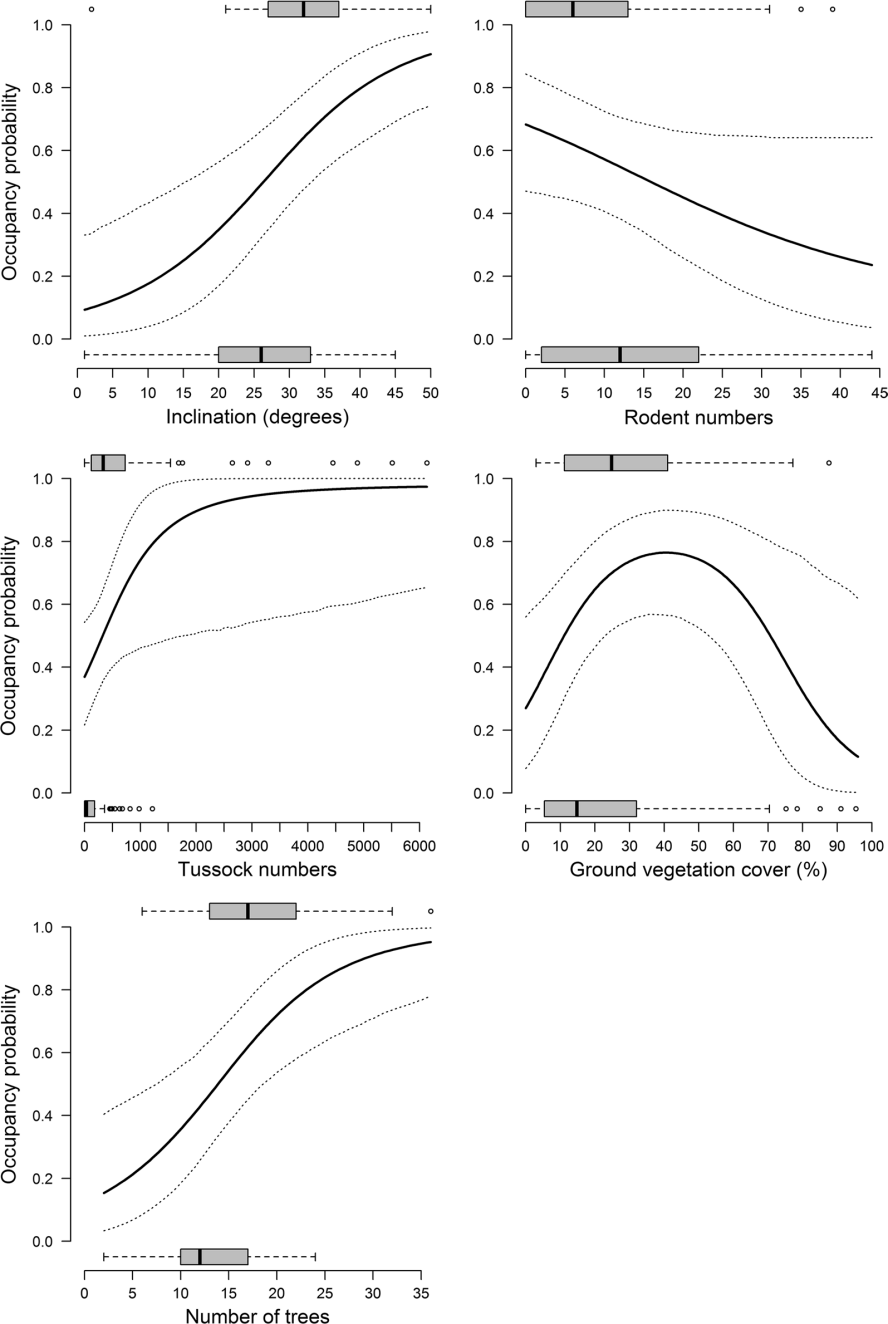
Habitat variables discriminating breeding territories and control areas. Shown are plots of the five variables whose model-averaged coefficients did not include 0 (cf. Table 3). The* solid lines* are fitted values based on model-averaged coefficients of the seven top-ranked GLMMs of the across-hypotheses analysis (Table 2), the* dotted lines* show 95 % confidence limits. To calculate the fitted values, the variable of interest (x-axis) was varied within the observed range while the others were fixed on their average values. For each variable,* inset box plots* show median (*bold line*), 25–75 % range (*grey box*), range of data within 1.5 times the interquartile range from the corresponding quartile (*whiskers*) and observations beyond this range (*dots*) for occupancy probability equaling 0 (control areas) or 1 (breeding territories). N_territories_ = 73, n_control areas_ = 73

According to model-averaging (Table 3a), territory occupancy of the wood warbler was positively related to *number of tussocks*, *number of trees* and *slope steepness* (Fig. 4). On the other hand, territory occupancy showed a quadratic relationship with *cover of herb layer* and was negatively related to *rodent numbers*. *Number of bushes*, *tree dbh* and the quadratic effect of *tree species diversity*, the other three variables included in some of the five top models, appeared to be less important in explaining territory occupancy, as the 95 % confidence intervals of their estimates included 0 (Table 3a).

**Table 3:** Estimates, standard errors (SE), and 2.5–97.5 % confidence limits based on model-averaging from the across-hypotheses model selection of (A) breeding territories vs. control areas (n = 73 pairs) and (B) breeding territories (n = 56) vs. abandoned territories (n = 20)

Hypothesis	Variable	Estimate	SE	2.5 %	97.5 %
(a) Forest structure					
Ground variables	Cover of herb layer (lin. term)	0.98	0.45	0.09	1.87
	Cover of herb layer (quad. term)	−0.71	0.28	−1.25	−0.16
	Number of bushes	−0.58	0.37	−1.31	0.15
	Number of tussocks	1.78	0.81	0.18	3.37
Tree variables	Number of trees	0.94	0.31	0.32	1.55
	Tree dbh	−0.48	0.31	−1.09	0.13
	Tree species diversity (lin. term)	−0.22	0.27	−0.75	0.31
	Tree species diversity (quad. term)	0.27	0.21	−0.13	0.67
Topography	Slope steepness	0.91	0.28	0.35	1.46
Rodent-avoidance	Rodent numbers	−0.60	0.30	−1.19	−0.01
(b) Forest structure					
Tree variables	Number of trees	1.72	0.70	0.36	3.09
Disturbance	Distance to forest edge	3.58	1.52	0.60	6.57
Topography	Slope steepness	2.20	0.76	0.71	3.68

Shown are variables included in the highest ranking models and in models with ΔAICc < 2 to the highest ranking ones

*Lin* linear; *quad* quadratic

### Breeding vs. abandoned territories

#### Interactions with year (analysis step 1)

In the forest structure hypothesis, subgroup ground variables, one model including the interaction of *number of tussocks* and *year* ranked highest (ΔAICc to second-best model = 6.35 and to null model = 13.62), and this interaction was retained for the next step. For all other hypotheses (and subgroups), models including interactions with *year* received no support, and interactions with *year* were not further considered.

#### Within-hypothesis analysis (analysis step 2)

##### Forest structure hypothesis

Subgroup ground variables—The highest-ranking model included the interaction between *number of tussocks* and *year* along with the respective main effects (ΔAICc to null model: > 12, Table 4). Four other models were within ΔAICc < 2 to the best model. All included *number of tussocks* and, in various combinations, the interaction between *number of tussocks* and *year*, *number of bushes* (linear and quadratic effects) and *cover of herb layer*. We thus retained *number of tussocks,* its interaction with *year*, *number of bushes* (linear and quadratic effects) and *cover of herb layer* (linear effect) for the across-hypotheses analysis.

**Table 4:** Model selection results of the analysis of breeding territories (n = 56) vs. abandoned territories (n = 20)

Hypothesis	Variables in model	LL	K	AICc	ΔAICc	Weight
Forest structure						
(a) Ground variables	Number of tussocks, year, number of tussocks x year	−22.315	7	60.277	0	0.202
	Number of tussocks, number of bushes^2^	−22.774	7	61.194	0.917	0.128
	Number of tussocks, number of bushes^2^, cover of herb layer	−21.562	8	61.273	0.996	0.123
	Number of tussocks, year, number of tussocks x year, number of bushes	−21.798	8	61.745	1.468	0.097
	Number of tussocks, year, number of tussocks x year, cover of herb layer	−21.986	8	62.122	1.845	0.080
	…					
	Null	−31.868	4	72.299	12.022	0.000
(b) Tree variables	Number of trees^2^, tree dbh^2^, tree species diversity^2^	−17.092	10	57.569	0	0.091
	Number of trees, tree species diversity^2^	−21.187	7	58.020	0.452	0.072
	Number of trees, tree dbh^2^, tree species diversity^2^	−18.652	9	58.031	0.462	0.072
	Number of trees^2^	−22.514	6	58.246	0.677	0.065
	Number of trees, tree species diversity^2^, tree dbh	−20.060	8	58.269	0.701	0.064
	Number of trees, tree dbh^2^, tree species diversity^2^, sky visibility	−17.695	10	58.776	1.207	0.050
	Number of trees^2^, tree dbh^2^, tree species diversity^2^, sky visibility	−16.336	11	58.797	1.229	0.049
	Number of trees^2^, tree species diversity^2^	−20.364	8	58.877	1.308	0.047
	Number of trees^2^, tree dbh^2^, tree species diversity^2^, sky visibility^2^	−15.234	12	59.420	1.852	0.036
	…					
	Null	−31.868	4	72.299	14.730	0.000
(c) Tree species composition	Proportion conifers	−29.723	5	70.303	0	0.265
	Proportion conifers, proportion beech	−29.533	6	72.283	1.981	0.098
	Proportion conifers, proportion other deciduous trees	−29.539	6	72.295	1.992	0.098
	…					
	Null	−31.868	4	72.299	1.996	0.098
Rodent-avoidance	Null	−31.868	4	72.299	0	0.575
	Rodent numbers	−31.023	5	72.903	0.605	0.425
Disturbance	Distance to forest edge, distance to path^2^	−20.719	7	57.084	0	0.694
	Distance to forest edge^2^, distance to path^2^	−20.453	8	59.055	1.97	0.259
	…					
	Null	−31.868	4	72.299	15.214	0.000
Topography	Slope steepness^2^, elevation^2^, southness, eastness	−13.541	10	50.467	0	0.692
	…					
	Null	−31.868	4	72.299	21.832	0.000
Across hypotheses^a^	Slope steepness, distance to forest edge, number of trees	−14.985	7	45.457	0	0.734
	…					
	Null	−31.868	4	72.299	26.842	0.000

For each hypothesis, the top-ranked model (ΔAICc = 0), the models with ΔAICc < 2 to the top-ranked model and the null model (referred to as “null”) are shown. “…” refers to additional models examined, but not listed in detail to avoid overlong table

*LL* log-likelihood; *K* number of parameters in the model (including intercept), *weight* Akaike weight (chance of the model to be the best one, given the candidate models)

The quadratic effect of a variable x, composed of a linear and a quadratic component (x ± x^2^), is denoted as x^2^

Each model included x- and y-coordinates (and their interaction) of territories to account for spatial autocorrelation

^a^ Only linear terms of variables from best models per hypothesis and at most three habitat variables jointly used due to convergence problems with quadratic terms and more than three habitat variable per model

Subgroup tree variables—Best-supported models consistently included *number oftrees*, either as linear or quadratic effect (Table 4). Likewise, the quadratic effects of *tree species diversity* and *tree dbh*, respectively, were included in the best-supported model and in most models with ΔAICc < 2 to the best one. Finally, the linear or the quadratic effect of *sky visibility* was included in a few models with ΔAICc < 2 to the best model. We thus retained *number of trees* and *tree dbh* (for both variables as linear and quadratic effects) as well as *tree species diversity* (quadratic effect) and *sky visibility* (linear and quadratic effects) for the across-hypotheses analysis.

Subgroup tree species composition variables—*Proportion conifers* was included in the best-supported model. Log-likelihood values of two other supported models (ΔAICc < 2 to the best one) and the best-supported model were very similar, suggesting that the inclusion of *proportion beech* or *proportion other deciduous trees* did not contribute to explaining variation in the data (Table 4). We thus only retained the variable *proportion conifers* for the across-hypotheses analysis.

##### Rodent-avoidance hypothesis

The rodent-avoidance hypothesis received no support, because the top-ranked model was the null model (Table 4). We thus did not retain the variable *rodent numbers* for the across-hypothesis analysis.

##### Anthropogenic disturbance hypothesis

The best-supported model (ΔAICc to null model: 22.96, Table 4) included the linear effect of *distance to forest edge* and the quadratic effect of *distance to path*. One other model had a ΔAICc < 2 to this model and included the quadratic effects of both *distance to forest edge* and *distance to path*. The linear and the quadratic effects of *distance to forest edge* and the quadratic effect of *distance to path* were kept for the across-hypotheses analysis.

##### Topographic hypothesis

The highest-ranked model included the quadratic effects of *slope steepness* and *elevation* and the linear effects of *southness* and *eastness*, respectively (ΔAICc to null model: >21, Table 4). All other models had ΔAICc > 2.5 to the highest-ranking one. We retained the quadratic effects of both *slope steepness* and *elevation* and the linear effects of *southness* and *eastness* for the across-hypotheses analysis.

#### Across-hypotheses analysis (analysis step 3)

Because *rodent numbers* was not identified as relevant in step 2, interactions between *rodent numbers* and habitat variables were not analysed. The combined analysis of the variables, which were retained from the high-ranking models of the hypothesis-specific analyses above, was problematic because many models of the across-hypotheses analyses had convergence problems. The problematic models always included the interaction between *number of tussocks* and *year*. We thus simplified the analysis by dropping this interaction and including instead *number of tussocks* as linear effect, and by including variables from only the top-ranked model per hypothesis. Because this simplification did not alleviate the convergence problems, we continued by only using linear effects instead of quadratic effects and by jointly including at most three habitat variables (plus the x- and y-coordinates and their interaction, see “Methods” section) in the different models. In the 176 candidate models based on the ten variables *distance to forest edge*, *number of tussocks*, *number of trees*, *slope steepness*, *elevation*, *aspect *(via *southness* and *eastness* jointly), *distance to path*, *tree species diversity*, *tree dbh* and *proportion conifers*, convergence problems no longer occurred.

ΔAICc of the highest-ranked model to the null model was 26.8. No other model was within ΔAICc 2 of the highest-ranked one (Table 4), which included *slope steepness*, *number of trees* and *distance to forest edge*.

**Fig. 5:**
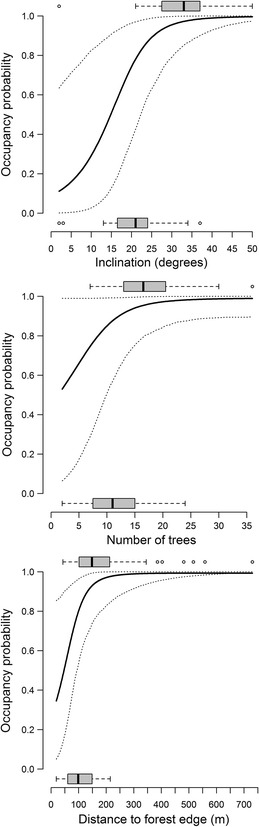
Habitat variables discriminating breeding and abandoned territories. Shown are *plots* of the three variables whose model-averaged coefficients did not include 0 (Table 3). The *solid lines* are fitted values based on model-averaged coefficients of the three best-supported GLMs of the across-hypotheses analysis (Table 4), the* dotted lines* show 95 % confidence limits. To calculate the fitted values, the variable of interest (x-axis) was varied within the observed range while the others were fixed on their average values. For each variable, inset box plots show median (*bold line*), 25–75 % range (*grey box*), range of data within 1.5 times the interquartile range from the corresponding quartile (*whiskers*) and observations beyond this range (*dots*) for occupancy probability equaling 0 (abandoned territories) or 1 (breeding territories). N_occupied_ = 56, n_abandoned_ = 20

Model-averaging across all models revealed that *slope steepness*, *number of trees* and *distance to forest edge* were the variables for which 95 % confidence intervals of their estimates did not include 0 (Table 3b). Thus, the topography hypothesis (via the variable *slope steepness*), the forest structure hypothesis (via *number of trees*) and the anthropogenic disturbance hypothesis (via *distance to forest edge*) were supported (Fig. 5).

## Discussion

### Forest structure hypothesis

Forest structure in terms of both the ground vegetation (*number of tussocks*, *cover of ground vegetation*, *number of bushes*) and the tree layer (*number of trees* and *tree dbh*) was important for the selection of breeding territories in the wood warbler. Collectively, our findings suggest that wood warblers preferred to set up territories in forest stands of medium age (25–75 % quartiles of *number of trees* and *tree dbh*, respectively, in breeding territories: 520–880 trees/ha and 22–30 cm, Table 1), corresponding to late pole wood as described in [38]. In the same study area as ours, but using remote sensing data, wood warbler territories were shown to be located in stands with relatively even-aged trees of medium height and low vertical diversity of canopy height [39]. Such stands are characterized by a relatively closed canopy and an open stem space with little branching below the canopy, resulting in relatively sparse ground (grass) vegetation cover and few bushes, i.e. little forest regeneration.

Our findings corroborate previous reports about associations of wood warblers with forest structure [5, 7–9, 11, 12, 40, 41]. Even though a preference for forests with a fairly closed canopy was not found in our study, median values for canopy closure were 87, 86 and 89.5 % in breeding territories, control areas and abandoned territories, respectively (estimated from sky visibility, Table 1) and thus within the range of values found in previous studies [60–90 %; e.g., 5, 9–11]. That canopy closure (i.e. sky visibility) did not differ between breeding territories, control areas and abandoned territories is in contrast to [39] who found canopy cover between 10 and 20 m above ground to be larger in breeding territories than in control areas in the same study area. These divergent results might be explained by the different methods used for assessing canopy closure. We measured canopy closure via an index of sky visibility (photographic camera pointed upwards) that included the foliage of all trees below the canopy while [39] assessed canopy closure between 10 m and 20 m height based on lidar signals.

Notwithstanding this methodological issue, wood warblers appear to favour forests with relatively little structural vertical diversity below a fairly closed canopy, as the number of bushes and young trees (dbh < 8 cm) was substantially lower in the breeding territories than in the control areas (Table 1). Aside from offering suitable conditions for nesting (see below), a relatively open under- and mid-storey may be particularly conducive to the wood warbler’s courtship behaviour, which includes song-flights from low branches between tree trunks [7]. Note that openness in the under- and mid-storey does not simply arise from reduced tree density, as the number of trees (dbh > 8 cm) was lower in both control areas and abandoned territories than in breeding territories (Table 1). Fewer trees in a forest stand allow light to better penetrate the forest which in turn promotes growth of bushes and young trees, thereby reducing openness in the under- and mid-storey.

Breeding territory occupancy showed a quadratic relationship to ground vegetation cover (Fig. 4), corroborating previous findings about a preference for sparse ground vegetation typically around 20–30 % cover [5, 7, 9, 11]. Breeding territories also harboured markedly more grass and sedge tussocks than control areas and abandoned territories (Table 1). The occurrence of patches of ground vegetation cover appears to play a crucial role in the nesting ecology of the wood warbler. In Białowieża, 88.5 % of 156 nests were concealed among low (5–20 cm high) vegetation and under branches or spruce trees lying on the ground [49]. In our study, 87.7 % of 220 nests found between 2010 and 2014 were located in or very close to tussocks (own unpublished data). Not surprisingly, daily survival of wood warbler nests increased with nest concealment and with number of tussocks in our study areas [42].

An emergent property might arise from the combination of forest age structure and topographic conditions. On the one hand, relatively dense middle-aged forest stands situated on steep slopes might allow more direct sun radiation to reach the ground, favouring grasses and sedges, compared to similar forest stands in flat terrain. In steep forests, there is an increased probability of perpendicular incidence of sun rays onto the ground due to the spatial arrangement of trees. Canopy structure is in general measured vertically, either from above (lidar) or from the ground (photographic camera, this study), although most sun radiation is not vertical but diagonal. Interestingly, [39] found a positive relationship between wood warbler occurrence and potential direct solar radiation in March in the same study area. On the other hand, leaf litter might have a lower probability to accumulate on steep forest floors, inducing shallower soils and creating advantageous growth conditions for grasses and sedges compared to flat floors. This peculiar combination of features might explain the positive relationship of wood warbler breeding territories and inclination found in comparisons of breeding territories to both control areas and abandoned territories.

### Rodent-avoidance hypothesis

Breeding territory occupancy was strongly and inversely related to rodent numbers. This agrees with findings from [2, 3], both showing that across years local wood warbler numbers were significantly negatively correlated to rodent numbers. Our study furthermore suggests that rodents might also influence wood warbler habitat selection at a much smaller, within-forest-stand scale. An explanation for the avoidance of areas with many rodents could be that wood warblers aim at reducing the probability of nest predation. Nest success in wood warblers has been found to range from 34 % [6] to 59 % [43], and nest predation typically accounts for the majority of nest losses (80–95 % in Białowieża, [6]; 37 % in Wales, [44]; 79 % in our study population, [42]). However, direct predation by rodents appears to be rare (own unpublished data based on nest cameras) or not existent [44]. In Białowieża, nest loss rate was not related to rodent numbers, while the probability of nest failure was increased in rodent outbreak years [2, 6]. In our study area, nest survival was not related to rodent numbers either [42]. It thus seems that the avoidance of areas with many rodents is not due to direct predation of nests by rodents. Direct predation by rodents has been confirmed and implicated in territory selection in some other ground-breeding passerines (e.g., veerie *Catharus fuscescens* [19]; dusky warblers *Phylloscopus fuscatus* [18]). A more likely explanation for the avoidance of areas with many rodents could thus be that high prey densities attract rodent predators, thereby increasing the likelihood of (accidental) predation on wood warbler nests.

Breeding territories did not differ from abandoned ones in terms of rodent numbers, which rules out the possibility that territories were no longer occupied because of high rodent numbers. Thus, rodent-avoidance does not seem to be the reason for territory abandonment in our study area.

### Topography hypothesis

According to the comparison of breeding territories and control areas, wood warblers prefer to settle in relatively steep terrain, such as forested slopes, as already evidenced [e.g., 5, 7, 26]. Steep forested slopes primarily occur along valley sides in our study area. Wood warblers also settle at little inclined slopes in otherwise largely flat wooded terrain elsewhere [26]. Reasons for the preference for steep slopes can be manifold. First, suitable habitat structure, particularly in terms of ground vegetation cover, could be more likely to occur at inclined than flat areas (see the emergent property mentioned above). Second, reduced or absent forest management in steep terrain due to unfavourable conditions for economic exploitation of timber would result in more extensive forest stands, i.e. more suitable wood warbler habitat than in the heavily managed lowland forests. Third, disturbance from recreational activities is likely to be reduced on less accessible steep slopes. In fact, all our study areas with wood warbler occurrence were relatively remote, which not only reduces human recreational disturbance but also represents an obstacle to timber exploitation, with the last intensive harvesting carried out 20–50 years ago [38] depending on study site. Finally, as nest entrances in wood warblers are oriented horizontally, nests on slopes, with entrances facing away from the slope, could allow wood warblers to easily escape from nests without getting entangled in the vegetation [45].

Breeding territories were also located on steeper slopes than abandoned ones. Even though we do not have data on forest structure prior to abandonment, abandoned territories were not located in marginal habitats as indicated by the patterns of wood warbler occurrence before abandonment. More likely, territories in flatter terrain have become abandoned due to structural changes owing to forest management, resulting in the lower tree density compared to still occupied territories along slopes. Another reason for the abandonment of territories located in flatter areas might be the absence of the emergent property described earlier on: steep slopes provide more opportunities for sun radiation to reach the forest floor, while there is less leaf litter accumulation on steep forest floor, which both may promote ground vegetation cover. In the end, a conjunction of different factors may play a role in breeding habitat selection by wood warblers: more appropriate habitat structure, less disturbance by humans and possibly predators, and less detrimental timber exploitation.

### Anthropogenic disturbance hypothesis

Many studies have addressed the impact of disturbance by human recreational activities on wildlife, but general conclusions are hard to draw [46]. For instance, in grassland and forest habitats some species avoid the proximity of trails, while others, especially generalists, are attracted [20]. The influence of trails on breeding bird communities can be either due to increased edge effects, direct human disturbance, higher penetration of habitat by domestic and wild predators, or a combination thereof. In forests, it is especially ground-nesting species that seem to be negatively affected by disturbing visitors, increased predation and habitat change [22]. Under these premises, the wood warbler as a non-generalist, ground-nesting species is likely to be susceptible to disturbance [24].

The comparison of breeding territories vs. abandoned territories might indeed provide support for the disturbance hypothesis, but the difficulty remains to disentangle anthropogenic from predatory and/or habitat structure effects. Abandoned territories were located closer to the forest edge than current breeding territories (Fig. 5), while the two territory types did not differ in terms of distance to path or trails. Avoidance of edge-habitat could serve to reduce potential disturbance from increased human activities. However, avoidance of edge-habitat might equally likely result from increased presence of nest predators such as domestic cats or foxes, creating a “landscape of fear” [47], which appears to be widespread in wildlife [14, 15]. Finally, avoidance of edge-habitat could also be related to changes in habitat structure close to forest edges [e.g., 48], due to, for instance, altered light or microclimatic conditions promoting growth of under- and mid-storey vegetation, making the habitat unsuitable for wood warblers. Whether human recreational activities, predation pressure and/or habitat conditions close to forest edges have recently changed in our study area cannot be answered yet.

## Conclusions

Identifying and ranking cues associated to habitat selection, and linking these cues to fitness and population dynamics is critical for conserving and promoting high-quality habitats for threatened species. This study and a previous one [42] suggest, first, that grass and sedge tussocks are key habitat features for the wood warbler, affecting both territory selection and reproductive performance. Any forestry intervention promoting this type of ground vegetation is thus likely to enhance habitat quality for this declining passerine. Second, wood warblers prefer habitats with relatively high tree numbers, few bushes and an intermediate ground vegetation cover, for example in the form of tussocks. As these conditions are typically encountered in forest stands of middle age (i.e. pole wood), the wood warbler might be described as a coloniser of the middle stages of forest succession. However, the species also occurs in more mature woodland, such as old-growth forests [49], if they provide the structural features required (sparse and low-growing ground vegetation cover, relatively open stem space with few shrubs and bushes below a fairly closed canopy). Tree species do not appear to be decisive, as long as the required habitat structures are present [7; this study; T. Wesołowski pers. comm.].

Exploited forests might occasionally offer suitable conditions for the wood warbler, provided that management favours a high number of middle-aged trees that lead to stands with relatively closed canopy [39] and sufficient open space between tree trunks. In this sense, the current widespread small-scale thinning practice, which consists of removing few trees [i.e. single-tree selection and group-felling, 50] and favours light-demanding species, is clearly detrimental to the wood warbler as it creates too many gaps in the canopy. This practice boosts under-storey growth, particularly on fertile soils, and, due to competition, suppresses ground cover vegetation, especially grass and sedge tussocks.

In the mid-term, clear-felling of larger forest patches with subsequent re-growth leading to even-aged high forest stands would likely be more beneficial for the wood warbler than the current forestry practices that aim either at bringing more light into woodland by selective (and at times excessive) thinning, generating forest stands permanently covered with shrubs, bushes and trees of various sizes (continuous cover forestry), or at letting trees grow to climax in forest reserves. In the short-term, removal of shrubs and bushes in forest stands otherwise deemed suitable in structure for the wood warbler might perhaps provide temporary habitat for this endangered species. Future studies should more closely address (1) how forest structures suitable to the wood warbler can be achieved through targeted forest management, accounting for prevailing site conditions; (2) what are the mechanisms underlying avoidance of edge habitat (disturbance, predation, vegetation structure); and (3) why forest stands with low rodent densities are preferred by this European species.

## Additional files

10.1186/s12936-016-1298-2 Description of study areas. Study areas with coordinates, elevation, size, density and sample sizes for the comparisons of breeding territories vs. control areas (data from 2010 to 2012) and breeding territories vs. abandoned territories (data from 2010 and 2011).
10.1186/s12936-016-1298-2 Spearman rank correlations among habitat variables. Coefficients of Spearman rank correlations among habitat variables.
10.1186/s12936-016-1298-2 Modelling steps. Description of the three modelling steps (also see Fig. 3).
10.1186/s12936-016-1298-2 Model selection results. Model selection results of the analyses of breeding territories vs. control areas and breeding territories vs. abandoned territories when using the variables number of trees <4 m and number of trees <10 m, respectively, instead of number of trees. All three variables are highly correlated (see Additional file 2).

## Abbreviations

GLMM: generalized linear mixed-effects model
AICc: Aikake’s information criterion corrected for small sample size
