# Supplementary material for: Rodent-avoidance, topography and forest structure shape territory selection of a forest bird
Source: BMC Ecol. 2016 May 9;16:24. doi: 10.1186/s12898-016-0078-8 (PMC4860761; doi:10.1186/s12898-016-0078-8)
Supplement: Supplementary file 1 — 10.1186/s12936-016-1298-2 Description of study areas. Study areas with coordinates, elevation, size, density and sample sizes for the comparisons of breeding territories vs. control areas (data from 2010 to 2012) and breeding territories vs. abandoned territories (data from 2010 and 2011). [file 12898_2016_78_MOESM1_ESM.pdf]

Table S1. Study areas with coordinates (N / E), elevation (m a.s.l.), size (km^2), territory density (SD) and sample sizes for the comparisons of breeding territories (B1) vs. control areas (C) (data from 2010 to 2012) and breeding territories (B2) vs. abandoned territories (A) (data from 2010 and 2011).

Study area	Coordinates	Elevation[1]	Size	Density[2]	B1	C	B2	A
Belchen	47°21.7’ / 7°48.6’	1022	0.18	16.67 (-)	3	3	3	0
Bänkerjoch	47°26.2’/ 8°2.1’	682	1.15	2.63 (0.00)	1	1	1	0
Blauen	47°27.6’ / 7°31.7’	683	2.21	4.41 (3.06)	4	4	3	0
Erschwil	47°22.5’ / 7°33.2’	712	1.28	5.47 (-)	2	2	0	0
Ennenda	47°2.3’ / 9°5.0’	642	0.74	6.76 (1.35)	11	11	7	0
Gündelhardt	47°38.7’ / 8°56.4’	543	1.33	0.75 (-)	1	1	1	5
Hochwald	47°27.4’ / 7°39.7’	711	1.06	3.77 (-)	3	3	3	0
Homberg	47°21.5’ / 7°51.1’	861	1.19	2.1 (1.78)	5	5	4	0
Kleinlützel	47°26.3’ / 7°25.9’	627	0.85	8.85 (0.9)	11	11	7	3
Langenbruck	47°21.3’ / 7°47.0’	991	0.67	5.83 (3.54)	3	3	3	0
Lauwil	47°22.5’ / 7°39.7’	980	0.52	12.74 (6.23)	9	9	9	3
Montsevelier	47°22.1’ / 7°29.5’	674	2.35	1.67 (0.9)	9	9	5	0
Mönthal	47°30.5’ / 8°8.6’	559	0.55		0	0	0	3
Oltingen	47°25.8’ / 7°56.5’	732	0.44	0.36 (-)	1	1	1	0
Scheltenpass	47°20.8’ / 7°37.1’	927	1.14	6.43 (5.06)	8	8	8	0
Staffelegg	47°25.4’ / 8°4.1’	620	1.75	0.83 (0.00)	2	2	1	0
Ueberstorf	46°52.2’ / 7°20.8’	736	0.29	-	0	0	0	3
Wintersingen	47°30.8’ / 7°50.1’	517	0.68	-	0	0	0	3
Total			16.86	5.07 (4.36)	73	73	56	20

[1] For Mönthal, Ueberstorf and Wintersingen averaged over abandoned (A) territories, for all other study areas averaged over breeding territories (B1) and control territories (C).

[2] Average density of territories (pairs with or without nests) per km^2 per study area across the three study years. Missing SD (-) due data from one year only.
