# Supplementary material for: Rodent-avoidance, topography and forest structure shape territory selection of a forest bird
Source: BMC Ecol. 2016 May 9;16:24. doi: 10.1186/s12898-016-0078-8 (PMC4860761; doi:10.1186/s12898-016-0078-8)
Supplement: Supplementary file 2 — 10.1186/s12936-016-1298-2 Spearman rank correlations among habitat variables. Coefficients of Spearman rank correlations among habitat variables. [file 12898_2016_78_MOESM2_ESM.pdf]

Coefficients of Spearman rank correlations among habitat variables. Values above the diagonal refer to the data comparing breeding territory (n = 73) vs. control areas (n = 73), values below the diagonal refer to the data comparing breeding territories (n = 56) vs. abandoned territories (n = 20). Correlations > |0.7| are printed in bold. For a description of variables, see Table 1. Abbreviations of column names are given below the table.

	Ele	Slo	Exp	Dfe	Dpa	Sky	Chl	Tus	Bus	Ntr	T<4	T<10	Dbh	Div	Rod	Odt	Bee	Con
**Ele**	1	0.431	-0.017	-0.159	0.163	-0.198	-0.325	0.130	-0.343	0.215	0.261	0.320	-0.140	0.030	-0.416	-0.113	0.150	0.062
**Slo**	0.506	1	-0.154	-0.067	0.124	-0.142	-0.136	0.218	-0.129	0.118	0.172	0.260	-0.144	0.080	-0.074	-0.016	0.116	-0.087
**Exp**	-0.390	-0.063	1	-0.080	0.095	0.060	0.018	-0.006	-0.146	0.022	-0.047	-0.006	0.207	-0.005	-0.224	0.021	-0.017	0.009
**Dfe**	-0.043	-0.086	0.163	1	0.129	0.217	0.057	0.086	-0.044	0.012	-0.006	-0.020	-0.040	0.013	0.054	0.088	-0.072	-0.005
**Dpa**	0.081	0.050	0.059	0.135	1	0.112	-0.015	0.137	-0.102	0.176	0.185	0.197	-0.115	0.131	-0.089	-0.053	-0.088	0.180
**Sky**	-0.105	-0.023	0.134	0.207	0.078	1	0.378	0.174	0.233	-0.241	-0.155	-0.226	0.108	0.077	0.022	0.146	-0.217	-0.010
**Chl**	-0.062	-0.026	0.220	0.113	0.112	0.322	1	0.585	0.343	-0.067	-0.011	-0.102	-0.053	0.231	0.144	-0.050	-0.060	0.207
**Tus**	0.306	0.220	0.020	0.262	0.238	0.114	0.402	1	0.044	0.179	0.217	0.249	-0.262	0.334	-0.060	-0.055	-0.078	0.285
**Bus**	-0.228	0.010	0.060	-0.170	-0.222	0.126	0.346	0.013	1	-0.285	-0.142	-0.237	0.032	0.164	0.368	0.001	-0.040	0.099
**Ntr**	0.138	0.223	0.186	0.086	0.044	-0.209	0.000	0.197	0.011	1	**0.791**	**0.868**	-0.502	0.362	-0.008	-0.136	-0.040	0.301
**T<4**	0.245	0.193	0.122	0.048	0.144	-0.096	0.054	0.286	0.003	**0.784**	1	**0.901**	-0.639	0.333	-0.032	-0.226	0.021	0.323
**T<10**	0.294	0.259	0.097	0.066	0.071	-0.170	-0.021	0.291	-0.008	**0.899**	**0.918**	1	-0.568	0.324	-0.045	-0.197	0.058	0.287
**Dbh**	-0.133	-0.063	-0.030	-0.120	-0.142	0.056	-0.066	-0.233	0.071	-0.655	**-0.744**	-0.676	1	-0.355	0.036	0.010	0.120	-0.259
**Div**	0.034	0.169	0.316	0.062	0.240	0.023	0.267	0.336	0.135	0.477	0.371	0.429	-0.363	1	0.152	-0.035	-0.266	0.662
**Rod**	-0.074	-0.052	0.044	0.082	0.008	-0.058	-0.044	0.041	-0.036	0.210	0.143	0.194	-0.066	0.205	1	-0.190	0.004	0.190
**Odt**	-0.153	-0.041	-0.055	-0.035	-0.033	0.279	0.045	-0.014	0.096	-0.275	-0.306	-0.306	0.019	0.046	-0.203	1	-0.650	-0.415
**Bee**	0.141	0.064	-0.015	-0.039	-0.118	-0.339	-0.155	-0.066	-0.197	0.033	0.036	0.051	0.086	-0.283	0.100	**-0.755**	1	-0.282
**Con**	0.108	0.006	0.201	0.074	0.308	-0.009	0.286	0.347	0.121	0.425	0.447	0.457	-0.272	0.664	0.225	-0.399	-0.149	1

Ele = Elevation; Slo = Slope steepness; Exp = Exposition; Dfe = Distance to forest edge; Dpa = Distance to path; Sky = Sky visibility; Chl = Cover of herb layer; Tus = Number of tussocks; Bus = Number of bushes; Ntr = Number of trees; T<4 = Number of trees branched < 4 m; T<10 = Number of trees branched < 10 m; Dbh = Tree dbh; Div = Tree species diversity; Rod = Rodent numbers; Odt = Prop. other decid. Tree; Bee = Prop. Beech; Con = Prop. conifers.
