# Supplementary material for: Rodent-avoidance, topography and forest structure shape territory selection of a forest bird
Source: BMC Ecol. 2016 May 9;16:24. doi: 10.1186/s12898-016-0078-8 (PMC4860761; doi:10.1186/s12898-016-0078-8)
Supplement: Supplementary file 3 — 10.1186/s12936-016-1298-2 Modelling steps. Description of the three modelling steps (also see Fig. 3). [file 12898_2016_78_MOESM3_ESM.pdf]

Modeling steps

Step 1: Assessing the relevance of interactions of habitat variables with year

For each of the four hypotheses, we first evaluated the importance of the interactions between habitat variables and year (Figure 3, main text). Here, the candidate model set for each hypothesis consisted of a null model only including the intercept and the random effects (only breeding territories vs. control areas) and the intercept and the x- and y-coordinates plus their interaction (breeding territories vs. abandoned territories), respectively, a model with all the habitat variables per hypothesis (for the forest structure hypothesis: with all the habitat variables per subgroup), and as many additional models as there were habitat variables, with each additional model including *year* as a fixed, categorical effect and the interaction of one habitat variable with *year* in addition to all the habitat variables per hypothesis (subgroup). As an example, the candidate model set for the subgroup “ground variables” of the forest structure hypothesis consisted of five models: 1) the null model, 2) one model with the three habitat variables *cover of herb layer*, *number of tussocks*, *number of bushes*, 3) model 2 plus *year* and the interaction between *year* and the first habitat variable, *cover of herb layer*, 4) model 2 plus *year* and the interaction between *year* and the second habitat variable, *number of tussocks*, and 5) model 2 plus *year* and the interaction between *year* and the third habitat variable, *number of bushes*. Interactions between habitat variables and year were deemed relevant, if included in models judged to have considerable support (ΔAICc < 2 compared to the best model), and carried to the next analysis step.

Step 2: Within-hypothesis analysis

Here, we evaluated candidate models consisting of all possible combinations of habitat variables per hypothesis (and within subgroups of the forest structure

hypothesis), including the interaction(s) with *year* identified as important in the previous step (but no interactions among the habitat variables). An interaction (e.g. *rodent numbers* x *year*) and the fixed effect *year* were always entered to or removed from a model as a group; that is, *year* never occurred in a model without an interaction. In addition, quadratic effects were examined for all habitat variables to check for simple curvilinear relationships. Model comparison was done as described above based on AICc.

Step 3: Across-hypotheses analysis

To jointly address potential effects of habitat variables from different hypotheses, variables from the top-ranked model and from models with ΔAIC < 2 to the top-ranked model per hypothesis identified as outlined in step 2 were considered. We first examined the relevance of interactions of each structural and topographic habitat variable with rodent numbers to see whether interactions needed to be included in the final across-hypothesis modelling step. Interactions with rodent numbers were considered because rodent numbers can strongly fluctuate across years. The candidate model set consisted of a null model including the intercept and the random effects (breeding territories vs. control areas) and the intercept and the x- and y-coordinates plus their interaction (breeding territories vs. abandoned territories), respectively, then for each habitat variable two models, of which one model included the respective habitat variable and *rodent numbers* and the other model the two main effects plus their interaction. As an example, for the habitat variable *number of tussocks*, one model consisted of the main effects *number of tussocks* and *rodent numbers*, the second model included the main effects and the interaction between *number of tussocks* and *rodent numbers.* Interactions between habitat variables and *rodent numbers* were deemed relevant, if included in the best-supported model or in

models with ΔAICc < 2 to the best one and then carried to the final analysis step. Here, models consisting of all possible combinations of habitat variables were compared, including quadratic effects and/or interactions with *year* and *rodent numbers,* respectively, where relevant. Model comparison was done as described above via AICc. In addition, effect sizes, associated standard errors and 95% confidence intervals were obtained by model-averaging over all candidate models.
