# Supplementary material for: Rodent-avoidance, topography and forest structure shape territory selection of a forest bird
Source: BMC Ecol. 2016 May 9;16:24. doi: 10.1186/s12898-016-0078-8 (PMC4860761; doi:10.1186/s12898-016-0078-8)
Supplement: Supplementary file 4 — 10.1186/s12936-016-1298-2 Model selection results. Model selection results of the analyses of breeding territories vs. control areas and breeding territories vs. abandoned territories when using the variables number of trees <4 m and number of trees <10 m, respectively, instead of number of trees. All three variables are highly correlated (see Additional file 2). [file 12898_2016_78_MOESM4_ESM.pdf]

Model selection results of the analysis of a) breeding territories vs. control areas (n = 73 pairs) and b) breeding territories vs. abandoned territories (n = 56 pairs) for the variables *number of trees < 4 m* and *number of trees < 10 m* (forest structure hypothesis, subgroup tree variables; see main text for results concerning the other hypotheses). Model selection was carried out separately for these two highly correlated variables. The top-ranked model (ΔAICc = 0), the models with ΔAICc < 2 to the top-ranked model and the null model (referred to as "null") are shown. "…" refers to additional models examined, but not listed in detail to avoid overlong table, as they were little informative. LL = log-likelihood, K = number of parameters in the model (including random effects and intercept), weight = Akaike weight (chance of the model to be the best one, given the candidate models). The quadratic effect of a variable x, composed of a linear and a quadratic component ($x \pm x^2$), is denoted as x^2.

Hypothesis	Variables in model	LL	K	AICc	ΔAICc	Weight
a)						
- trees < 4 m	number of trees branched < 4 m, tree dbh	-91.501	5	193.430	0	0.116
	tree dbh	-92.604	4	193.492	0.062	0.112
	number of trees branched < 4 m, tree dbh^2	-91.382	6	195.368	1.938	0.044
	tree dbh^2	-92.492	5	195.413	1.983	0.043
	…					
	null	-101.199	3	208.568	15.138	0.000
- trees < 10 m	number of trees branched < 10 m, tree dbh	-89.077	5	188.582	0	0.155
	number of trees branched < 10 m, tree dbh, tree species $diversity^2$	-87.604	7	190.019	1.437	0.075
	…					
	null	-101.199	3	208.568	15.138	0.000

Hypothesis	Variables in model	LL	K	AICc	ΔAICc	Weight
b)						
- trees < 4 m	number of trees branched < 4 m^2, tree dbh^2, tree species $diversity^2$	-18.533	10	60.451	0	0.401
	…					
	null	-31.868	4	72.299	11.847	0.001
- trees < 10 m	number of trees branched < 10 m^2, tree dbh^2, tree species $diversity^2$	-18.946	10	61.276	0	0.112
	number of trees branched < 10 m, tree dbh^2, tree species $diversity^2$	-20.416	9	61.559	0.283	0.097
	number of trees branched < 10 m, tree species $diversity^2$	-22.996	7	61.638	0.363	0.093
	number of trees branched < 10 m, tree dbh^2, tree species $diversity^2$, sky visibility	-19.632	10	62.649	1.374	0.056
	number of trees branched < 10 m^2, tree dbh^2, tree species $diversity^2$, sky $visibility^2$	-17.048	12	63.048	1.772	0.046
	number of trees branched < 10 m^2, tree dbh^2, tree species $diversity^2$, sky visibility	-18.465	11	63.055	1.780	0.046
	…					
	null	-31.868	4	72.299	11.023	0.000

Across-hypothesis, breeding territories vs. control areas: Using either *number of trees branched < 4 m* or *number of trees branched < 10 m* resulted in models with considerable less support compared to models with *number of trees* (see main text).

Across-hypothesis, breeding territories vs. abandoned territories: Using either *number of trees branched < 4 m* or *number of trees branched < 10 m* resulted in models with almost identical support compared to models with *number of trees*.
